# Is Non-Stimulated C-Peptide at Diagnosis a Good Predictive Value for Insulin Use at Two Years after Diagnosis in Pediatric Diabetic Patients?

**DOI:** 10.3390/medicina57090902

**Published:** 2021-08-29

**Authors:** Wei-Chih Chou, Yen-Yin Chou, Yu-Wen Pan, Meng-Che Tsai

**Affiliations:** 1 Division of Genetics, Endocrinology, and Metabolism, Department of Pediatrics, National Cheng Kung University Hospital, College of Medicine, National Cheng Kung University, Tainan 704, Taiwan; penguin031077@hotmail.com (W.-C.C.); yenyin@mail.ncku.edu.tw (Y.-Y.C.); panyuwen0527@hotmail.com (Y.-W.P.); 2 Department of Pediatrics, Hualien Tzu Chi Hospital, Buddhist Tzu Chi Medical Foundation, Hualien 970, Taiwan

**Keywords:** non-stimulated C-peptide, glutamic acid decarboxylase (GAD) antibodies, diabetes mellitus (DM), glucagon test

## Abstract

*Background and Objectives*: Insulin treatment may be initially required to stabilize patients presenting with metabolic crisis at type 1 and 2 diabetes mellitus (DM) onset. Some patients with type 2 DM may need persistent insulin treatment. This study aimed to examine the predictive performance of non-stimulated C-peptide level at the time of diagnosis for future insulin use in pediatric diabetic patients. *Materials and Methods*: We reviewed the medical charts of diabetic patients aged 18 years or younger in a medical center in southern Taiwan from January 2000 to December 2019. Clinical and individual data were collected at the time of DM diagnosis. Outcomes were persistent insulin use at the time of diagnosis, as well as at one and two years after diagnosis. *Results*: The final analysis included a total of 250 patients. The best cut-off point of non-stimulated C-peptide was 0.95 ng/mL, and the predictive indices for the insulin use were 0.84 for sensitivity and 0.94 for specificity at two years after DM diagnosis. Incorporating age at onset and presence of GAD antibodies can further increase the predictive power of non-stimulated C-peptide. *Conclusions*: The value of non-stimulated C-peptide at diabetic onset was feasible and effective for predicting future insulin treatment up to the time point of two years after diagnosis.

## 1. Introduction

Type 1 diabetes mellitus (T1DM) results from autoimmune-mediated destruction of the insulin-producing β-cells, and thus, T1DM patients may require long term insulin treatment [1]. Its pathogenesis is different from type 2 diabetes mellitus (T2DM), which is characterized by increased insulin resistance. However, insulin treatment may be initially required to stabilize patients presenting with metabolic crisis in both types. After stabilization, insulin therapy is usually adjusted according to serum glucose levels in the follow-up period, where insulin can be tapered off in T2DM cases [2,3]. In Taiwan’s health care system, T1DM is recognized as a catastrophic illness that requires long-term health care and more psychosocial support. T1DM patients’ medical expenditure can be reimbursed once their diagnosis is documented. Therefore, patients and their family are keen to know whether long term insulin therapy is required for this disease.

Serial serum C-peptide levels in a glucagon stimulation test are one of the important indicators of residual β-cell function, and these levels may be associated with choice of therapy [4,5]. However, repeated blood withdrawals sometimes are difficult and painful to children. As such, whether a single test for non-stimulated C-peptide is predictive of insulin use in the years after diabetic onset remains interesting and unaddressed in children. In adults, T2DM patients with negative islet autoantibodies and preserved C-peptide levels defined by a fasting level > 0.99 ng/mL are likely to retain endogenous β-cell function at 1 year, and they can achieve glycemic control without insulin [6]. One previous study investigated the utility of glucagon-stimulated C-peptide levels in differentiating T1DM and T2DM diagnosis [5]. However, little is known, as of yet, about the predictive role of a single non-stimulated C-peptide level at the time of diagnosis in future insulin use. 

Therefore, this study aimed to examine the predictive performance of non-stimulated C-peptide level for insulin use in pediatric diabetic patients. Specifically, we sought to determine the optimal cutoff of non-stimulated C-peptide levels for predicting insulin use at and one and two years after diabetic onset. Classification and regression tree (CART) analysis is used to classify subjects into two categories of insulin use that are designed to handle numbers of predictor variables [7].

## 2. Materials and Methods 

### 2.1. Study Subjects

**Figure 1:**
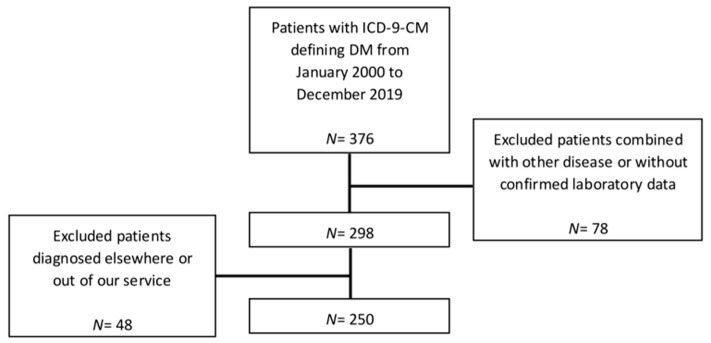
The flowchart for the selection of patients; DM: diabetes mellitus; ICD-9-CM: International Classification of Diseases, Ninth Revision, Clinical Modification.

We reviewed the medical charts of pediatric patients aged 18 years or younger with a diagnosis of diabetes mellitus in a medical center that received referrals from a catchment area of nearly 3 million residents in southern Taiwan. Our review retrospectively found a total of 376 cases coded with International Classification of Diseases, Ninth Revision, Clinical Modification (ICD-9-CM) code 250 that defines diabetes mellitus from January 2000 to December 2019. Among them, 78 cases were excluded due to other underlying diseases (such as leukemia) or lacking confirmed laboratory data, and 48 other cases were excluded due to missing pre-treatment or follow-up data because they were diagnosed elsewhere or transferred out of our service after diagnosis. The entire procedure was approved by the Institutional Review Board of the Cheng Kung University Hospital (A-BR-107-033, approved on 24/10/2018). The final analysis included a total of 250 patients (Figure 1).

### 2.2. Collection of Laboratory Data

Medical records were reviewed to obtain relevant clinical data of diabetic patients, including serum levels of insulin and C-peptide, lipid profiles, liver enzymes and creatine, as well as glutamic acid decarboxylase (GAD) antibodies and urine or serum keto acids. Diagnosis of diabetes mellitus (DM) was defined by random plasma glucose levels ≥ 200 mg/dL, fasting glucose levels ≥ 126 mg/dL or glycated hemoglobin (HbA1c) ≥ 6.5%. We also measured the body mass index (BMI) of patients at DM onset. BMI was calculated by dividing weight in kilograms by height in square meters. We determined the Z-score of BMI according to the gender and age-specific BMI charts using the Taiwan children and adolescent growth chart published in 2010 [8]. For the analytic purpose, patients were divided into two age groups with the age of DM onset dichotomized into above and under age 10 years. Likewise, patients with obesity defined by a BMI Z-score greater than 2 were compared those with a BMI Z-score less than 2.

### 2.3. Statistical Analysis

Outcome variables of interest were insulin use at the time of DM diagnosis and at time points of 1 and 2 years after DM diagnosis. In order to determine the predictive value of non-stimulated C-peptide levels at DM diagnosis for insulin use, we used receiver operating characteristic (ROC) analyses to identify the best cutoff value based on the maximum of [sensitivity + (1-specificity)] under the area under curve (AUC). Herein the non-stimulated status included fasting or random samples.

Once the cutoff value of non-stimulated C-peptide levels was determined, univariate and multivariate logistic regression analyses, with insulin use as the outcome variable, were applied to determine the predictability of potential predictors. We examined the predictive role of gender, age of DM onset, BMI at DM onset, non-stimulated insulin and C-peptide levels, GAD antibodies, and ketoacidosis to verify whether the abovementioned parameters are effective in predicting future insulin use [9]. In multivariate regression analysis, stepwise predictor selection was used with a significance level of 0.05 set for entry and 0.1 for stay in the model. Odds ratios (ORs) with 95% confidence interval (CI) were reported for the predictors which remained in the final model. 

Next, in order to generate the optimal prediction model for future insulin use, classification and regression tree (CART) analysis was applied to develop recursive partitioning that classified subjects into various risk groups. CART analysis could delineate the complex interactions among variables in the final tree, rather than simply identifying the interactions in a multivariable logistic regression model. The Hosmer–Lemeshow test was further used to determine the model fit.

## 3. Results

Table 1 described the parameters among a total of 250 diabetic patients (122 males, 48.8%) at the time of diagnosis. The average age at onset was 9.69 (±4.39) years with a BMI Z-score of 1.05 (±2.65). The positive rate of GAD antibodies at DM onset was 58%, and 64% of patients presented with ketoacidosis at the time of diagnosis. There was no gender difference in the demographic and clinical parameters. 

**Figure 2:**
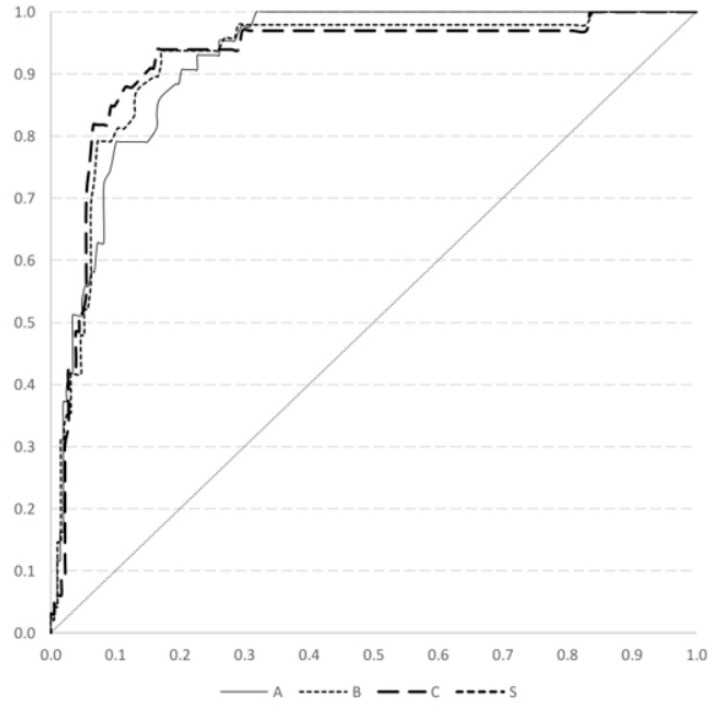
The receiver operating characteristic analysis for the association between non-stimulated levels of C-peptide and insulin use at the time of diabetes mellitus onset (Line A), one year (Line B) and two years (Line C) after diagnosis.

We calculated the area under curve (AUC) in the ROC analysis investigating the relationship between non-stimulated C-peptide and insulin use (Figure 2). The AUC was 0.92 (95% CI: 0.89–0.96, *p* <0.001) for insulin use at DM onset, 0.92 (95% CI: 0.88–0.97, *p* < 0.001 for one year after DM diagnosis, and 0.92 (95% CI: 0.87–0.98, *p* < 0.001) for two years after DM diagnosis. According to our previously mentioned criteria, the best cut-off point of non-stimulated C-peptide was 0.95 ng/mL, while the predictive indices for insulin use were 0.8 for sensitivity and 0.92 for specificity at DM diagnosis, 0.83 for sensitivity and 0.92 for specificity at one year after DM diagnosis, and 0.84 for sensitivity and 0.94 for specificity at two years after DM diagnosis (Table 2). Therefore, we used this value to dichotomize our patients in the following analysis. 

In univariate regression analysis, we found several factors, including age under 10 years, BMI Z-score less than 2, non-stimulated C-peptide level less than 0.95 ng/mL, the presence of GAD antibodies, and ketoacidosis, to be associated with insulin use at three different time points (Table 3). Particularly, non-stimulated C-peptide level less than 0.95 ng/mL was the most salient factor associated with insulin use. In multivariate logistic regression analyses, only age, non-stimulated C-peptide level, and GAD antibodies remained significantly associated with insulin use in the final model. Again, non-stimulated C-peptide level was the only predictor of insulin use consistently found at three time points, i.e., at DM diagnosis, and one and two years after diagnosis. 

**Figure 3:**
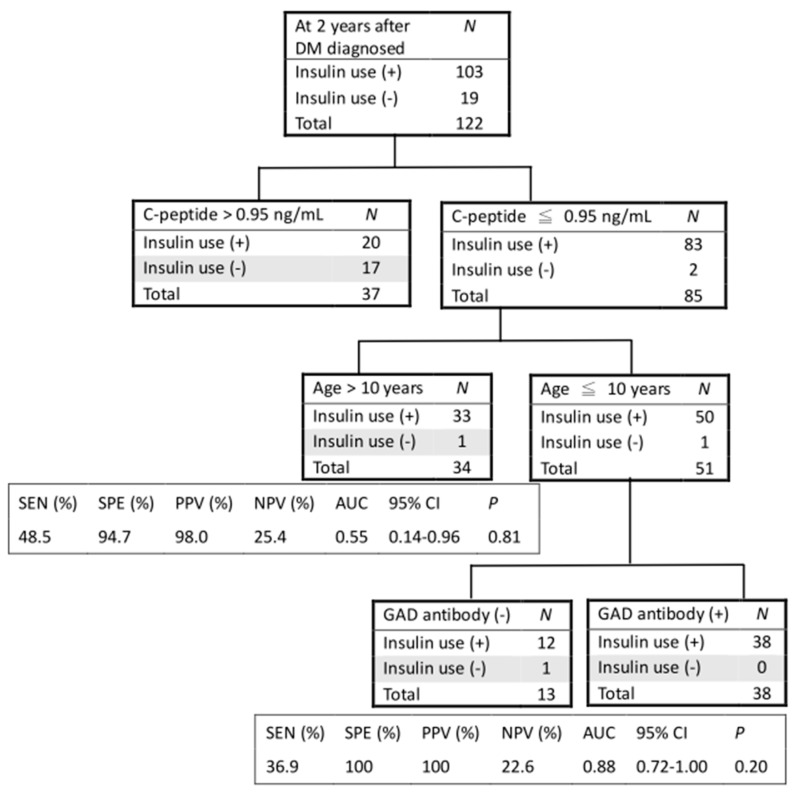
CART analysis at 2 years after DM diagnosis to predict insulin use. For each node in the tree, the numbers of insulin use are displayed. SEN: sensitivity; SPE: specificity; PPV: positive predictive value; NPV: negative predictive value; AUC: area under the curve; CI: confidence interval; DM: diabetes mellitus; GAD: glutamic acid decarboxylase.

Figure 3 presents the CART analysis in predicting the insulin use at two years after DM diagnosis. The positive predictive value (PPV) for insulin use was 0.98 and the specificity was 0.95 if a patient with an age under 10 had a non-stimulated C-peptide level less than 0.95 ng/mL. Furthermore, the positive predictive value and specificity both reached 1 if a patient with an age under 10 had a non-stimulated C-peptide level less than 0.95 ng/mL and presence of GAD antibodies.

## 4. Discussion

Our analysis clearly demonstrated the predictive performance of non-stimulated C-peptide level at the time of diagnosis in determining future insulin use up to two years after diagnosis. We found that a cut-off value of non-stimulated C-peptide at 0.95 ng/mL was the most salient factor associated with insulin use at two years after diagnosis, with a positive predictive value of nearly 99%. When planning future health care for pediatric diabetic patients, a single measurement of non-stimulated C-peptide at diagnosis can be a feasible and reliable test to predict insulin use in clinical routines.

The role of C-peptide, which is usually used as an indicator of β-cell function, in clinical practice has been implicated in assisting classification of diabetic patients. Prior research in adults has shown that a glucagon-stimulated C-peptide level of 0.9–1.8 ng/mL could be used to differentiate insulin-requiring from non-insulin-requiring diabetes, and patients with a glucagon-stimulated C-peptide level < 0.2 nmol/L (0.6 ng/mL) might be likely to have an absolute requirement for insulin [10,11]. There is less evidence in children, and a higher cut-off value may be needed to make a differential diagnosis between insulin-dependent and non-insulin-dependent DM. Only one previous study investigated the clinical utility of the six minute glucagon stimulation test in evaluating β-cell function among pediatric diabetic patients [5]. Tung et al. suggested cut-off values for fasting and post-glucagon C-peptide levels in distinguishing T1DM from T2DM to be 2.1 ng/mL and 3.3 ng/mL, respectively, with post-glucagon C-peptide levels having better discriminating power than fasting C-peptide levels [5]. Compared to Tung et al.’s findings, the cut-off value of non-stimulated C-peptide in this present study is much lower. We argue that the measurement of C-peptide took place at the time of diagnosis, when patients were often in a hyperglycemic status before initiating insulin therapy. Under such a circumstance, the C-peptide level may better reflect residual β-cell function as compared to those levels based on the results of glucagon stimulation tests. 

In addition to C-peptide levels, previous studies on adult patients showed that younger age at onset, lower values of BMI, elevated fasting plasma glucose levels, and presence of auto-antibodies were associated with earlier insulin initiation [9,12]. Similarly in our sample of pediatric patients, age at onset and the presence of GAD antibodies, along with levels of non-stimulated C-peptide, were the most salient predictors for insulin use based on multivariate logistic regression analyses. BMI was initially associated with insulin use in univariate analysis, but the significance of association was canceled in multivariate analysis. It is plausible that our patients were young in age, such that severe insulin resistance had not been developed. Meanwhile, fasting plasma glucose level at onset may not be a reliable predictor, as it might be associated with multiple factors, such as environmental, behavioral, and emotional factors [13]. Further incorporating age at onset and GAD antibodies in CART analysis significantly increased the predictive power of non-stimulated C-peptide. A diabetic patient with age at onset under 10 and having a non-stimulated C-peptide level less than 0.95 ng/mL and GAD antibodies was predicted to use insulin in two years’ time. These prognostic indicators are helpful in disease management and family counselling when seeing patients at the time of diagnosis. 

There are some limitations in our study. First, we did not obtain pubertal measurements in our patients, and thus were unable to conduct subgroup ROC analysis separately for pre-pubertal and pubertal patients, although we controlled for age at onset, which was arbitrarily dichotomized into above and under age 10 years in regression analyses. Prior research has indicated that pubertal diabetic patients may have higher fasting and post-stimulated C-peptide values than pre-pubertal peers in the six minute glucagon test [5]. Further research with a larger patient cohort may be needed to investigate age-specific residual β-cell function in pediatric diabetic patients. Second, our study only followed patients up to two years after diagnosis. Whether the status of insulin use changed after this time point remained unaddressed, such as in cases with slowly progressing T1DM. Lastly, the decision of initiating or tapering off insulin use was determined by clinical physicians who may have personal preferences for medication choices. Novel hypoglycemic agents, such as glucagon-like peptide-1 agonists, are continually being approved for pediatric patients and therefore postpone the initiation of insulin use in T2DM. Further prospective research may be required to verify our results. 

## 5. Conclusions

The level of non-stimulated C-peptide at diabetic onset was a feasible, effective, and less painful measurement for predicting future insulin treatment up to the time point of two years after diagnosis. Incorporating age at onset and presence of GAD antibodies can further increase the predictive power of non-stimulated C-peptide. Obtaining these prognostic data may help when guiding and advising patients and their parents regarding therapeutic plans.

## Figures and Tables

**Table 1:** Clinical parameters at the time of DM diagnosis; DM: diabetes mellitus; BMI: body mass index; GAD: glutamic acid decarboxylase. Positive rate of GAD antibodies and ketoacidosis calculated from positive numbers divided by the total numbers of tested people.

	Male	Female	Total
Number, *n* (%)	122 (48.8%)	128 (51.2%)	250
Age at DM onset	10.07 (±4.59)	9.32 (±0.17)	9.69 (±4.39)
BMI at DM onset (Z score)	1.47 (±3.14)	0.64 (±2.01)	1.05 (±2.65)
C-peptide (ng/mL)	1.29 (±1.66)	1.16 (±1.71)	1.22 (±1.69)
Insulin (uU/mL)	16.54 (±51.53)	11.46 (±19.40)	13.93 (±38.51)
GAD antibody-positive, *n* (%)	38 (51%)	50 (65%)	88 (58%)
Ketoacidosis, *n* (%)	66 (63%)	73 (64%)	139 (64%)

**Table 2:** Predictive values of different cut-off C-peptide levels for future insulin use. SEN: sensitivity; SPE: specificity; PPV: positive predictive value; NPV: negative predictive value; DM: diabetes mellitus.

C-Peptide Cut-Off Point	DM Onset				1 Year after DM Diagnosis				2 Years after DM Diagnosis			
ng/mL	SEN (%)	SPE (%)	PPV (%)	NPV (%)	SEN (%)	SPE (%)	PPV (%)	NPV (%)	SEN (%)	SPE (%)	PPV (%)	NPV (%)
≦0.9	78.7	90.7	97.6	47.0	82.4	93.8	98.1	57.0	82.4	93.9	98.7	49.2
≦0.95	79.7	90.7	97.6	48.1	82.9	91.7	97.6	57.1	83.5	93.9	98.7	50.8
≦1.0	83.1	86.0	96.6	51.4	86.5	87.5	96.5	61.8	87.4	87.9	97.5	55.8

**Table 3:** Univariate and multivariate regression analyses of the predictors of insulin use at different time points after diabetes diagnosis. Age was dichotomized to groups with age > 10 and age ≦ 10 years old; BMI Z-score was dichotomized to groups with ≦ 2 SD and > 2 SD; C-peptide was dichotomized to groups with values ≦ 0.95 and > 0.95 ng/mL; GAD antibodies were dichotomized to groups as yes and no; ketoacidosis was dichotomized to groups as yes and no. DM: diabetes mellitus; BMI: body mass index; GAD: glutamic acid decarboxylase.

	DM onset			1 Year after DM diagnosis			2 Years after DM diagnosis		
Univariate	Exp (B)	95% CI	*p*	Exp (B)	95% CI	*p*	Exp (B)	95% CI	*p*
Gender (male vs. female)	1.12	0.58–2.16	0.742	0.56	0.29–1.07	0.078	0.51	0.24–1.10	0.087
Age (≦10 years vs. >10 years)	8.87	3.76–20.90	<0.001	11.53	4.90–27.13	<0.001	10.33	3.80–28.03	<0.001
BMI Z score (≦2 SD vs. >2SD)	7.61	3.61–16.01	<0.001	12.45	5.88–26.38	<0.001	15.00	6.21–36.10	<0.001
C-peptide (value ≦ 0.95 vs. >0.95)	38.30	12.96–113.17	<0.001	53.33	17.93–158.63	<0.001	78.53	17.83–345.86	<0.001
Insulin level	1.01	1.00–1.01	0.255	1.03	1.01–1.05	0.008	1.02	1.00–1.04	0.072
GAD antibody (yes vs. no)	9.55	3.07–29.66	<0.001	21.44	6.09–75.46	<0.001	38.18	4.89–298.31	0.001
Ketoacidosis at onset (yes vs. no)	10.35	4.26–25.19	<0.001	6.68	3.09–14.47	<0.001	8.82	3.32–23.36	<0.001
**Multivariate analysis with stepwise selection**									
Age (≦10 years vs. >10 years)	6.28	0.73–54.10	0.094	4.49	0.81–24.97	0.086			
C-peptide (value ≦ 0.95 vs. >0.95)	15.37	3.19–73.98	0.001	12.93	2.46–67.78	0.002	26.13	2.97–229.84	0.003
GAD antibody (yes vs. no)				7.19	1.33–38.87	0.022	8.49	0.91–78.87	0.060

## Data Availability

The data are not publicly available due to regulations related to patient privacy in the consent.
